# A modified niche model for generating food webs with stage‐structured consumers: The stabilizing effects of life‐history stages on complex food webs

**DOI:** 10.1002/ece3.7309

**Published:** 2021-03-27

**Authors:** Etsuko Nonaka, Anna Kuparinen

**Affiliations:** ^1^ Department of Biological and Environmental Sciences University of Jyväskylä Jyväskylä Finland

**Keywords:** allometric trophic network, community dynamics, life‐history stage, multilayer network, ontogenetic shift, predator–prey interaction

## Abstract

1. Almost all organisms grow in size during their lifetime and switch diets, trophic positions, and interacting partners as they grow. Such ontogenetic development introduces life‐history stages and flows of biomass between the stages through growth and reproduction. However, current research on complex food webs rarely considers life‐history stages. The few previously proposed methods do not take full advantage of the existing food web structural models that can produce realistic food web topologies.
2. We extended the niche model developed by Williams and Martinez (*Nature*, 2000, *404*, 180–183) to generate food webs that included trophic species with a life‐history stage structure. Our method aggregated trophic species based on niche overlap to form a life‐history structured population; therefore, it largely preserved the topological structure of food webs generated by the niche model. We applied the theory of allometric predator–prey body mass ratio and parameterized an allometric bioenergetic model augmented with biomass flow between stages via growth and reproduction to study the effects of a stage structure on the stability of food webs.
3. When life‐history stages were linked via growth and reproduction, more food webs persisted, and persisting food webs tended to retain more trophic species. Topological differences between persisting linked and unlinked food webs were small to modest. The slopes of biomass spectra were lower, and weak interaction links were more prevalent in the linked food webs than the unlinked ones, suggesting that a life‐history stage structure promotes characteristics that can enhance stability of complex food webs.
4. Our results suggest a positive relationship between the complexity and stability of complex food webs. A life‐history stage structure in food webs may play important roles in dynamics of and diversity in food webs.

## Graphical abstract

We extended the niche model developed by Williams and Martinez (2000) to generate food webs that included trophic species with a life‐history stage structure. We then applied the theory of allometric predator–prey body mass ratio to parameterize an allometric bioenergetic model augmented with biomass flow between stages via growth and reproduction. When life‐history stages were linked, more food webs persisted and persisting food webs tended to retain more trophic species.

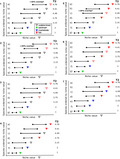

## 1 INTRODUCTION

A large body of research in the last several decades has investigated potential factors that can promote the structural and dynamical stability of complex food webs and their constituent populations. These factors include hierarchically ordered feeding (Williams & Martinez, 2000), characteristic predator–prey body mass ratios (Brose et al., 2006), allometric degree distributions of feeding links (Otto et al., 2007), compartmentalization (Stouffer & Bascompte, 2011), weak interaction links including weak omnivory (Gellner & McCann, 2016; Kratina et al., 2012; Stouffer & Bascompte, 2010) and reduced predation pressure at low densities ((Koen‐Alonso & Yodzis, 2005; Martinez et al., 2006; Williams & Martinez, 2004b), pairwise negative correlation between interaction strengths (Allesina et al., 2015; Tang et al., 2014), and self‐regulation (e.g., cannibalism, intraspecific interference, (Barabás et al., 2017; Rall et al., 2008), among others (reviewed by Brose & Dunne, 2010; Dell et al., 2005). More recently, studies have started incorporating different types of interactions in complex food webs (multiplex or multilayer networks; Fontaine et al., 2011; Kéfi et al., 2012, 2016) to account for multiple types of ecological interactions, such as mutualisms and parasitism, in which organisms simultaneously engage in natural communities.

A ubiquitous feature of natural systems is that almost all organisms grow in size during their lifetime and switch diets, trophic positions, species interacting with, and habitats as they grow (de Roos, 2020; Werner, 1986; Werner & Gilliam, 1984). Such ontogenetic development introduces life‐history stages and flows of biomass between the stages through growth and reproduction to food webs, collectively forming complex multilayer ecological networks. Studies have shown that ontogenetic diet shifts have far‐reaching effects on competitive and predator interactions, population dynamics, and community structure in small food web modules (Nilsson et al., 2018; Persson, 2002; de Roos & Persson, 2003). The persistence of consumers can be enhanced in life‐history structured communities through biomass overcompensation in consequence of ecological asymmetry between different stages (e.g., juveniles are better competitors than adults; Persson & de Roos, 2013). Such asymmetry, however, can also be expected to destabilize populations by inducing cohort cycles or alternative stable states without a predator (Persson & de Roos, 2013). Research on how these effects in small food web modules may scale up to an entire complex food web is still in its infancy, and so are the tools to generate life‐history structured complex food webs in a biologically justifiable manner.

Studies have reported the mixed effects of including a stage structure on the stability of complex food webs (Bland et al., 2019; Mougi, 2017; Rudolf & Lafferty, 2010). Rudolf and Lafferty (2010) found that, using static topological models of food webs, structural robustness to species removal was lower with a stage structure than without. They pointed out that species might be more sensitive to resource loss when ontogenetic stages were sequential resource specialists. Bland et al., (2019) used population dynamical models of complex food webs and showed that non‐stage‐structured food webs lost twice as many consumer taxa as stage‐structured webs, while the variability of biomass dynamics did not differ. Mougi (2017) also used similar population dynamical models and concluded that species persistence (the fraction of species persisting in a food web) increased as the proportion of stage‐structured species increased in the food webs and that the effect was more pronounced in food webs with a greater number of species and interactions. More studies are needed to elucidate the role of a stage structure on persistence and stability and how it may come about in complex food webs.

Rudolf and Lafferty (2010) and Bland et al. (2019) used the niche model (Williams & Martinez, 2000) to generate network topologies and split a node into stages to create a stage‐structured taxon (nodes represent taxa, and interacting taxa are connected by links in ecological networks). The niche model has a demonstrated ability to produce many observed structural properties of empirical food webs despite its simplicity (Stouffer et al., 2005; Williams & Martinez, 2000) and has been the most widely used food web structural model. Splitting a node, as in Rudolf and Lafferty (2010) and Bland et al. (2019), can nontrivially modify the food web topology generated by the niche model, likely compromising the desirable properties of the food webs. Therefore, it is unclear how realistic the modified food webs in these studies would still have remained after new nodes and links were added to incorporate a life‐history structure. Firstly, minimizing the alteration of the network topology generated by the niche model is desirable because the model is known to be capable of producing realistic food web topology (Williams & Martinez, 2000, 2008) and because food web data resolved to life‐history stages to verify the topology of food webs with stage‐structured taxa are currently very scarce. Secondly, the niche model generates a “trophic species,” which is a functional group defined to consist of one or more taxa (e.g., species, genus, ontogenetic stages) that share the same sets of predators and prey (Cohen et al., 1990; Havens, 1992). Life‐history stages of a species are distinguished for their distinct ecological roles, at least partly by their characteristics related to feeding, so that a life‐history stage can be considered as a whole trophic species (not a fraction of it). Based on these interpretations and the observation that ontogenetic diet shifts are widespread in nature (Werner & Gilliam, 1984), a plausible alternative approach is to instead group nodes generated by the niche model to assemble a stage‐structured taxon. This approach allows preserving mostly the original topologies of the food webs from the niche model. No study has investigated this approach before.

We took the node‐grouping approach to introduce a stage structure into complex food webs. Following Bland et al. (2019), we applied the allometric trophic network (ATN) model of biomass dynamics (Brose et al., 2006) to the stage‐structured food webs on which we linked stages by biomass flow via growth and reproduction. We motivated the food webs studied here from aquatic communities at temperate and northern latitudes. It is well established that consumer–resource interactions are hierarchically structured largely by body size in aquatic communities because of the indeterminate growth of fishes and gape‐limited predation (Brose et al., 2019; Woodward & Hildrew, 2002). We found that food webs with stage‐structured consumers persisted more often and supported a greater number of species than food webs with non‐stage‐structured consumers.

## 2 MATERIALS AND METHODS

### 2.1 Introducing life‐history stage‐structured consumers to the food webs generated by the niche model

We built on the original niche model developed by Williams and Martinez (2000; Box 1) and incorporated an additional algorithm to construct life‐history structured consumers by grouping trophic species based on the extent of overlap between feeding ranges. As organisms grow in size during their ontogeny, they experience changing competition, predation, and energetic demands and may shift diets to maintain positive growth and minimize mortality (Werner & Gilliam, 1984). Ontogenetic diet shifts among life‐history stages within a species are widely observed in nature (Werner, 1986; Werner & Gilliam, 1984), with diet ranges overlapping at various degrees (Einum & Kvingedal, 2011; Lima & Zollner, 1996; Persson, 1983; Rezsu & Specziar, 2006; Rudolf & Lafferty, 2010; Woodward & Hildrew, 2002). Diet overlap is negligible in the case of diet shifts associated with habitat shifts (e.g., riverine vs. marine) or metamorphosis (e.g., aquatic vs. terrestrial), nested when organisms add larger prey to the diet as they grow in size, or partially nested because smaller prey are successively dropped from diet for energetic or mechanical reasons (Werner & Gilliam, 1984; Woodward et al., 2005). We applied this concept to construct food webs with consumers with a life‐history stage structure.

BOX 1 **The niche model by Williams and Martinez (2000)**

The niche model requires the number of trophic species (the number of nodes, $S_{0}$) and connectance (the fraction of realized feeding interactions out of all potentially possible, C) as input parameters (Table 1). It hierarchically ranks species according to the “niche value,” $n_{i}$, randomly drawn from a uniform distribution and assigns a feeding range to each species as follows. The range size, $r_{i}$ (*i* is the index for taxa), is determined by first drawing a random variable, $\eta_{i}$, from a beta distribution calibrated to obtain the desired connectance and then multiplying $\eta_{i}$ by the niche value for $i\;(r_{i}=\eta_{i}n_{i})$. The center of the feeding range, *c_i_*, is randomly chosen from a uniform distribution in $\left[\frac{r_{i}}{2},n_{i}\right]$, and the range is then determined as $\left[c_{i}‐\frac{r_{i}}{2},c_{i}+\frac{r_{i}}{2}\right]$. Therefore, species with larger niche values tend to have larger feeding ranges. The ranges are set such that cannibalism is allowed (i.e., *n_i_* can fall in the range of *i*). All the taxa whose niche values fall in the feeding range of another are regarded as the prey of the latter. The taxa with no prey are identified as basal taxa (i.e., autotrophs). The taxon with the lowest niche values is designated as an autotroph. We discard disconnected webs, webs with connectance beyond a given tolerance level ($C_{\mathrm{error}}$), and webs with taxa not connected to a basal taxon.

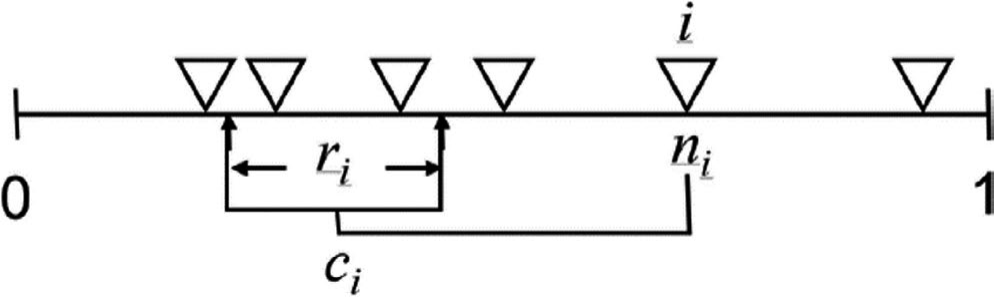

FIGURE. A schematic diagram showing how niche values and feeding range determine the trophic interactions. The niche values (indicated by upside‐down triangles) are ordered along the one‐dimensional niche space. The feeding range, *r_i_*, of taxon *i* is symmetric around the center, *c_i_*. Taxa whose niche values fall in the feeding range become the prey of taxon *i*. This figure is from Williams & Maritinez (2000).

**TABLE 1:** (a) The parameters, definitions, sources or remarks if relevant, and values used in the modified niche model to generate food webs. (b) The parameters, definitions, values used in this study, and sources or remarks if relevant, for the allometric trophic network (ATN) model

(a) Parameter	Definition	Value used	Sources or remarks	Values for sensitivity analysis (the number in the parentheses correspond to the number in Figure A5)
$S_{0}$	Number of species	60		
*C*	Connectance (proportion of realized links out of all possible)	0.15	Dunne et al. (2002); Bland et al. (2019)	
$C_{\mathrm{error}}$	Error tolerance on connectance	0.025	Bland et al. (2019)	
$N_{\mathrm{fishes}}$	The number of stage‐structured fish species	Between 2 and 6	Reasonable numbers of naturally cooccurring fish species in a community	
${\mathrm{Th}}_{\mathrm{fish}}$	A node at the trophic level ${>Th}_{\mathrm{fish}}$ can become a fish stage	2	The diet of fishes should include nonautotrophs (trophic level of pure herbivores is 2)	
${\mathrm{OL}}_{\min}$	Minimum overlap of niche ranges between consecutive stages of a fish	0.2	In terms of the fraction of the union of the two feeding ranges	0.1 (#2)
${\mathrm{Nstage}}_{\max}$	The maximum number of stages a fish species can have	5	Reasonable maximum numbers of fish stages	4 (#1)
${\mathrm{Nstage}}_{\min}$	The minimum number of stages a fish species should have	3	Reasonable minimum numbers of fish stages	2 (#1)

(b) Parameters	Definition	Value	Sources or remarks	Values for sensitivity analysis
*Z*	Body mass ratio	10^2.6^ for fish predators and prey 10^1.15^ for invertebrate predators and prey	Brose et al. (2006)	10^3.2^ (#3) and 10^2^ (#4) for fish, and 10^0.4^ (#5) and 10^1^ (#6) for invertebrates
*g_i_*	Autotroph intrinsic growth rate for *i*	Randomly drown from $0.8<N\left(0.9,0.5\right)<1$	Bland et al. (2019)	
K	Autotroph carrying capacity	540 μgC/L	Boit et al. (2012); Bland et al. (2019)	
*x_i_*	Mass specific metabolic rate of *i*	0 for autotrophs $0.314M_{i}^{‐0.15}$ for invertebrates $0.88M_{i}^{‐0.11}$ for fish	Bland et al. (2019); de Castro & Gaedke (2008); Killen et al. (2010, 2006)	
*y_ij_*	Maximum consumption rate of *i* eating *j*	4 for fish 8 for invertebrates	Brose et al. (2006); Boit et al. (2012)	
*e_ij_*	Assimilation efficiency of *i* eating *j*	0.95 when *j* is a fish 0.75 when *j* is an invertebrate 0.45 when *j* is an autotroph	Kelso (1972); Elliott (1976), Govoni et al. (1986); Yodzis and Innes (1992); Brose et al. (2006)	0.85 when *j* is a fish or an invertebrate (#7)
*f_m_*	Fraction of assimilated carbon respired for maintenance of basic bodily functions	0.05	Modified from Bland et al. (2019); Boit et al. (2012)	0.1 (*f_m_* and *f_a_* were varied simultaneously) (#8)
*f_a_*	Fraction of assimilated carbon that contributes to growth	0.5	Modified from Bland et al. (2019); Boit et al. (2012)	0.4 (*f_m_* and *f_a_* were varied simultaneously) (#8)
*q*	Hill exponent	1.5	A higher value than typically used in the ATN models (1.2–1.5) to achieve 10%–20% of food webs persisting after the ATN model is run (Martinez et al. 2006; Williams & Martinez, 2004b)	1.2 (#15) and 1.7 (#16)
$\omega_{\mathrm{ij}}$	Prey preference (relative to toward autotrophs)	When *i* is a fish, 500 times toward fishes 200 times toward invertebrates When *i* is an invertebrate, 100 times toward invertebrates More than toward autotrophs.	Fishes do not eat much autotrophs in temperate and northern regions (González‐Bergonzoni et al., 2012; Vejříková et al., 2016). Fish needs to eat fish to grow fast and large (Juanes et al., 2002; Post, 2003). The values are calibrated to achieve little consumption of autotrophs (mean 8%) by fishes with animal prey in their diets.	When *i* is a fish, 250 (#10) and 750 (#9) times toward fishes 200 times toward invertebrates When *i* is an invertebrate, 200 time toward invertebrates (#11), or 20 time toward invertebrates (#12)
$B_{o_{\mathrm{ij}}}$	Half saturation density of *i* eating *j*	1.5 μgC/L when an invertebrate *i* eats *j* 15 μgC/L when fish *i* eats fish *j* 20 μgC/L when fish *i* eats omnivore *j* 150 μgC/L when fish *i* eats small herbivores *j* (more than 50 times smaller than the fish in body mass) 15 μgC/L when fish *i* eats large herbivores *j* (not as much smaller than the fish in body mass)	(Bland et al. 2019; Martinez et al., 2012; Tonin, 2008) See Figure 1 in Bland et al., (2019) Herbivores are organisms whose diets consist of autotrophs for more than 70% (Bland et al. 2019).	
$c_{\mathrm{ij}}$	Consumer interference competition coefficient of *i* eating *j*	Randomly drawn from $0\leq \mathrm{Exp}\left(\lambda =5\right)\leq 0.5$ when k is an invertebrate $3\times 10^{‐4}$ when fish k eats fish j 10^−4^ when fish k eats omnivore j 1 when fish i eats small herbivore j 10^−4^ when fish k eats large herbivore j	Tonin, 2008; Martinez et al., (2012); Bland et al., (2019) See Figure 1 in Bland et al. (2019) All interspecific interference competition for feeding on prey j, $\left(c_{\mathrm{kj}}\right)$, is set to zero (i.e., $c_{\mathrm{kj}}=0$ for k≠i; intraspecific (within‐node) interference competition only).	Full interference competition (i.e., $c_{\mathrm{kj}}\neq 0$ when k≠i, #13), or intraspecific and inter‐fish stage interference competition (i.e., $c_{\mathrm{kj}}\neq 0$ when k and i are stages of the same trophic species, #14).
$p_{\mathrm{ij}}$	the fraction of resources of consumer i shared with consumer j	(the number of prey i shares with j)/the number of i's prey	Bland et al. (2019)	
I	Fraction of biomass invested to reproduction	0 for the first stage class 0.8 for the last stage class	Kuparinen et al. (2016) The increment between stages $=\frac{0.8}{\left(\mathrm{number}\;\mathrm{of}\;\mathrm{stages}‐1\right)}$	
$P_{\mathrm{mature}}$	Probability of reaching maturation at stage h,h=1,2,⋯,n	0 for the first stage class ${\left(1+e^{‐3\left(n‐n_{50}\right)}\right)}^{‐1}$ for higher stages, where $n_{50}=\frac{n}{2}$ is the stage at which 50% of the individuals become mature	Kuparinen et al. (2016)	
$a_{h}$	Fraction of biomass staying in the same stage	0.05 for h=1,⋯,n‐1 0.3 for h=n	Fish in the terminal stage reproduces without surplus energy and convert 30% of total biomass to offspring.	
$b_{h}$	Fraction of biomass moving to the next stage	$1‐a_{h}$		
L	Number of days in a growing season	90	Bland et al. (2019)	
${\mathrm{Th}}_{\mathrm{extinct}}$	Extinction threshold	10^−6^ μgC/L	When biomass goes below this value, the population is considered extinct	
${\mathrm{Th}}_{\mathrm{explode}}$	Maximum threshold	10^12^ μgC/L	When biomass exceeds this value, the population is no longer sustainable.	

**FIGURE 1:**
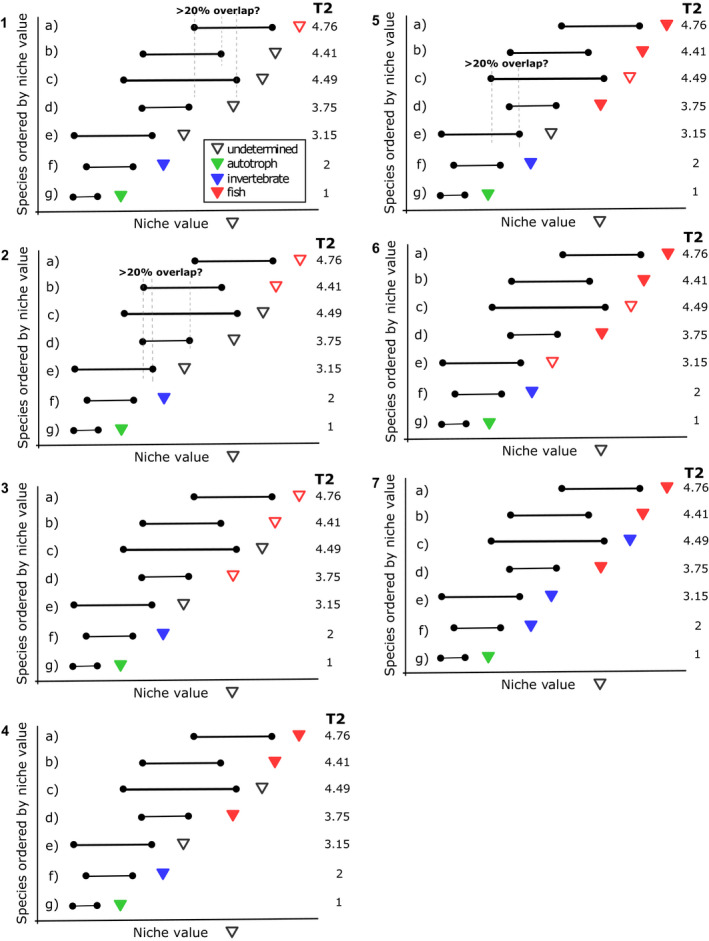
A highly simplified diagram showing how trophic species are classified and fish stages are assembled. A more detailed example is in Appendix A1. The upside‐down triangles indicate niche values of the seven nodes. T2, prey‐averaged trophic level, is calculated according to who eats whom in the entire community (the entire community is not shown here). (1) g is an autotroph because it has T2 = 1. f is an invertebrate because it has T2 = 2 (eats only autotrophs). a has the highest T2 and becomes the focal species (a fish candidate, indicated by an open red triangle). b and c have feeding ranges overlapping with that of a for more than 20% of the union of the two ranges and whose maxima fall in the range of a. (2) Because b's niche value is closer to a's, b is chosen as the next focal species. (3) Repeat the same procedure. d meets the conditions. (4) There are no species meeting the conditions for d in the rest of the community. Because we found 3 stages (the min number of stages is 3 in this example), we designate a, b, and d as a stage‐structured fish species. (5) c has the highest T2 in the remaining nodes and becomes the next focal species. Repeat the same procedure. (6) We find e to meet the conditions but fail to find another stage because we run out of nodes. (7) c and e instead become invertebrates. In this food web, there are one species of fish with three stages, 3 species of invertebrates, and 1 autotrophic species

**FIGURE 2:**
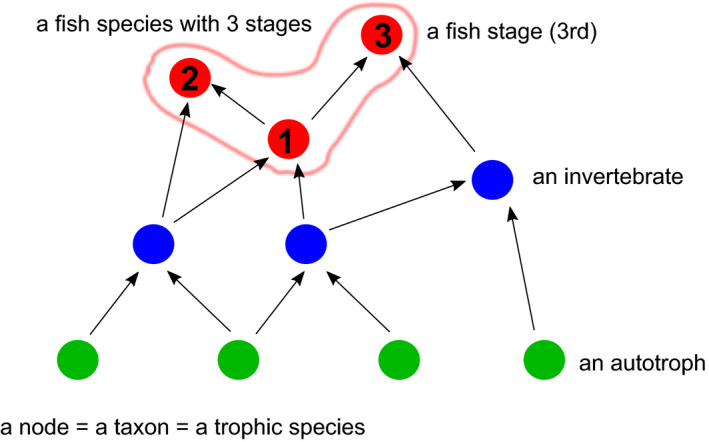
Clarification of terminology used in this paper. The figure shows a food web (feeding relationships) with 10 nodes and 12 links. In this paper, a node, a taxon, and a trophic species mean the same, and the terms are used interchangeably. A fish species is composed of multiple stages, each of which occupies a node. The numbers in the red dots indicate stages. In this figure, there are one fish species with three stages, 3 invertebrates, and 4 autotrophs

**FIGURE A1:**
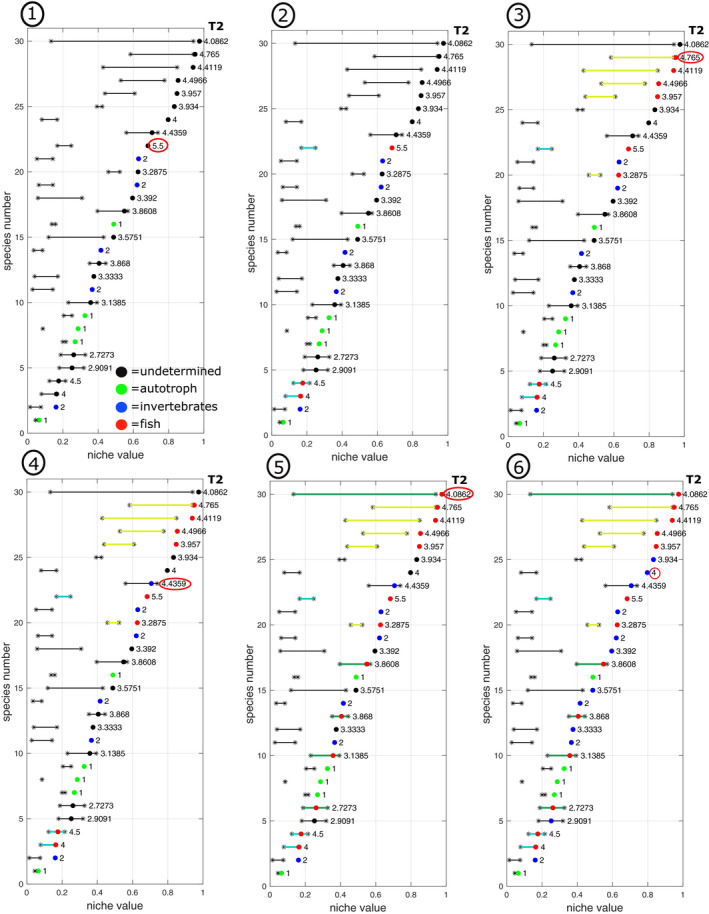
① T2 values are determined by the food web structure generated by the niche model. The niche values are indicated on the *x*‐axis. Remove autotrophs (T2 (prey‐averaged trophic level) = 1) and herbivores (T2 = 2) from the pool of fish candidates. Find the node with the highest T2 (#22 in this example). #22 becomes the focal node. ② Find nodes whose max of the feeding range falls in the feeding range of #22, whose range sufficiently overlaps with #22's range (20% in this example), and whose niche value is smaller than and the closest to #22's. #4 becomes the next focal node and repeat the steps until either the max number of stages are identified or no more nodes are available. In this example, #3 is the last one. #22, #4, and #3 form one stage‐structured population and are colored in turquoise. ③ #29 has the next highest T2. Identify other stages using the same procedure in 2. The stages of a new stage‐structured population are colored in yellow. ④ #23 has the next highest T2 but it fails to have a node with a sufficiently overlapping feeding range. #23 is classified as an invertebrate. ⑤ #30 has the next highest T2. #17, #13, #10, and #6 successively join the population. Because the max number of stages is 5 in this example, #5 will not join. ⑥ #26 has the next highest, but there are no more remaining nodes that meet the conditions. #21 and #19 are already assigned as invertebrates and not available to join. In fact, there are no other nodes that can satisfy the conditions. The remaining nodes are classified as invertebrates. The procedure is now completed

After obtaining food webs from the niche model, we assigned two measures of trophic position, the short‐weighted trophic level (*T*) and the prey‐averaged trophic level (*T*2), to each taxon (Williams & Martinez, 2000, 2004a, 2004b). The short‐weighted trophic level is the average of shortest trophic level (*T*1) and the prey‐averaged trophic level (Williams & Martinez, 2000, 2004a, 2004b). The shortest trophic level is equal to 1+ the shortest chain length from a basal species to the consumer, and the prey‐average trophic level is equal to 1+ the mean trophic level of all the consumer's resources (Williams & Martinez, 2004a, 2004b). An integer ($N_{\mathrm{fishes}}$; Table 1) was uniformly randomly drawn from an interval between the desired minimum and maximum numbers of stage‐structured taxa. We assumed that they were fishes (assume no stage structure in autotrophs and invertebrates) and that fishes fed on at least one nonbasal taxon (i.e., T2>2). To create a stage‐structured fish taxon, we first selected a species with the highest *T*2 (a “focal taxon”) that was greater than 2 (${\mathrm{Th}}_{\mathrm{fish}}$), indicating that this taxon ate at least one nonbasal taxon (Figure 1 and Figure A1 for an extended graphical example). We then identified taxa whose feeding range maxima fell within the range of the focal taxon with the overlap of the two feeding ranges greater than a specified minimum overlap (${\mathrm{OL}}_{\min}$) of the union of the two and whose niche value was smaller than and closest to the focal taxon's niche value, to become the next lower stage. This stage became the next focal taxon, and we repeated the steps until either the specified maximum number of stages (${\mathrm{Nstage}}_{\max}$) had been assigned or taxa whose range maxima fell in the range of the focal taxon with sufficient overlap ran out. When a focal taxon did not have any overlapping taxa to choose from to form the minimum number of stages (${\mathrm{Nstage}}_{\min}$), this taxon was disqualified and another taxon was chosen in the same way as the current focal taxon if other choices for the previous focal taxon were available. If it was impossible to find the minimum number of stages for the first focal taxon, it (but not the other ones that had been subsequently considered) was reclassified as a taxon without a stage structure and classified as an invertebrate. This occurred when a focal taxon happened to have a small feeding range so that no range optima fell in the range. The multiple taxa (nodes) selected in this procedure collectively made up one stage‐structured fish taxon and were removed from the pool of available taxa. We then chose another focal taxon with the next highest *T*2 from the remaining taxa and repeated the steps. We repeated these procedures until the chosen number of fishes had been created or taxa with suitable range overlaps had run out. If the minimum number of stage‐structured taxa could not be created, the food web was discarded. Predation of lower stages by higher stages within a stage‐structured taxon was interpreted as cannibalism. Cannibalism within a stage and cannibalism of higher stages by lower stages (very rare) were removed (cannibalism in nonstructured consumer taxa was kept). Taxa that were not identified as autotrophs nor fishes were identified as invertebrates (nonstructured consumers). Therefore, nodes in the network represented autotrophic trophic species, invertebrate trophic species, or life‐history stages of a fish. Hereafter, a node or a taxon refers to a non‐stage‐structured species (invertebrates and autotrophs) or a fish stage (Figure 2). A species refers to an autotrophic species, an invertebrate species, or a fish species that consists of three or more life‐history stages, while a stage refers to a fish life‐history stage.

### 2.2 Coupling stage‐structured food webs and biomass dynamics in the allometric trophic network (ATN) framework

To simulate deterministic population dynamics of the species, we employed a bioenergetic model in the allometric trophic network (ATN) framework developed by Brose et al. (2006) and expanded by Bland et al. (2019) to food webs with stage‐structured fishes (see Brose et al., 2006 for a complete description). Consequently, we used many parameter values and submodels used in their work.

#### 2.2.1 Body mass

**FIGURE A2:**
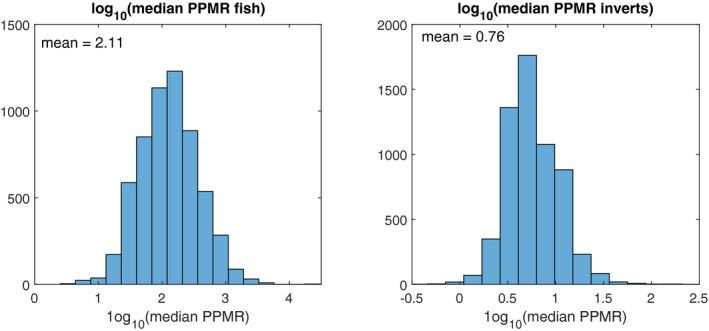
The distribution of median predator–prey body mass ratio for fish predators and invertebrate predators from the 30,000 original food webs. The means of the distributions are shown at the upper left corner. These distributions are within the empirical distributions shown in Figure 3 of Brose et al.'s (2006)

In this framework, body mass plays integral part in determining bioenergetic parameter values (Yodzis & Innes, 1992). More specifically, the rates of metabolism and maximum consumption are approximated by means of body mass scaling relationships (Yodzis & Innes, 1992). We calculated relative masses of the taxa based on the short‐weighted trophic position (*T*) in accordance with the theory of allometric predator–prey body mass ratio (Brose et al., 2006). We set the body mass ratio (*Z*) of fish predators and their prey to 10^2.6^ and of invertebrate predators and their prey to 10^1.15^ (Brose et al., 2006; Table 1b). The function, body mass, $M=\;Z^{T‐1}$, was used to define the body masses of invertebrates and the terminal stages of stage‐structured fishes. Hence, the body masses were relative to those of autotrophs whose body masses were defined to be equal to 1 (Bland et al., 2019; Brose et al., 2006). As in Bland et al. (2019), to model the well‐known pattern of fish growth with time, we used a von Bertalanffy isometric growth curve to define the body masses of lower stages (Table 2). We assumed that the individuals of terminal stages reach 90% of their asymptotic weight (Bland et al. 2019). Although body masses in lower stages no longer strictly conformed to the allometric body mass ratios, the median ratios from our model fell near the modes of the empirical distributions (Figure 3 in Brose et al., 2006; Figure A2).

**TABLE 2:** Equations for the model components in the ATN model

Model component	Formulation	Sources and notes	Values for sensitivity analysis
Body mass at stage h (The von Bertalanffy isometric growth curve)	$W_{h}=\;W_{\infty }{\left(1‐e^{‐K\left(h‐h_{0}\right)}\right)}^{3},$ where $K=\frac{3}{n}$ $h\;\in \left\{1,\cdots ,\;n\right\}$ v = terminal stage class of the fish $\left(\frac{W_{n}}{w_{\infty }}\right)=0.9$	Pauly (1980), Froese & Binohlan (2000), Bland et al. (2019) The value of $h_{0}$ is obtained by solving the equation for $h_{0}$ with = $W_{h}$0 and $W_{v}$ from the predator–prey body mass ratio	
The fraction of mature fish at each stage	$P_{\mathrm{mature}}=1\;/\;\left(1+e^{‐3\left(h‐h_{50}\right)}\right))$ *h*_50_ = the stage at which 50% of individuals are mature	Kuparinen et al. (2016) We assume $h_{50}$ occurs halfway through to the terminal stage	
Investment to reproduction	*I* = (*h* − 1)(*I*_max_/*n*) *I*_max_ = maximum investment = 0.2	Kuparinen et al. (2016)	
The Leslie matrix to model growth and reproduction by the terminal stage between growing seasons	${\left(\begin{array}{c} B_{i,1}\\ \begin{array}{c} B_{i,2}\\ \begin{array}{c} B_{i,3}\\ \begin{array}{c} \vdots \\ B_{i,n} \end{array} \end{array} \end{array} \end{array}\right)}_{t+1}=\left(\begin{array}{ccc} a_{1} & 0 & \begin{array}{cc} 0 & \begin{array}{cc} 0 & b_{v} \end{array} \end{array}\\ b_{1} & a_{2} & \begin{array}{cc} 0 & \begin{array}{cc} 0 & 0 \end{array} \end{array}\\ \begin{array}{c} \begin{array}{c} 0\\ 0 \end{array}\\ 0 \end{array} & \begin{array}{c} \begin{array}{c} b_{2}\\ 0 \end{array}\\ 0 \end{array} & \begin{array}{cc} \begin{array}{c} \begin{array}{c} a_{3}\\ \ddots \end{array}\\ 0 \end{array} & \begin{array}{c} \begin{array}{c} \begin{array}{cc} 0 & 0 \end{array}\\ \begin{array}{cc} \ddots & 0 \end{array} \end{array}\\ \begin{array}{cc} b_{n‐1} & a_{n} \end{array} \end{array} \end{array} \end{array}\right){\left(\begin{array}{c} B_{i,0}\\ \begin{array}{c} B_{i,1}\\ \begin{array}{c} B_{i,2}\\ \begin{array}{c} \vdots \\ B_{i,n} \end{array} \end{array} \end{array} \end{array}\right)}_{t}$ *b_h_* = the proportion of biomass in stage h to be shifted to stage h+1 (or to stage 1 for h=v), $a_{h}$ = the proportion of biomass in stage h to remain in the same stage $b_{n}$ = 0.3, $a_{n}=1‐b_{n}$ = 0.7	Modified from Bland et al. (2019)	*b_n_* = 0.5 (#17)
Stage‐specific harvesting sensitivity	$S_{\text{stage}}=\frac{1}{\left(1+e^{‐2\left(h‐n/2\right)}\right)},\text{for}h>1$ $S_{\mathrm{stage}}\left(h=1\right)=0$	Kuparinen et al. (2016)	

**FIGURE 3:**
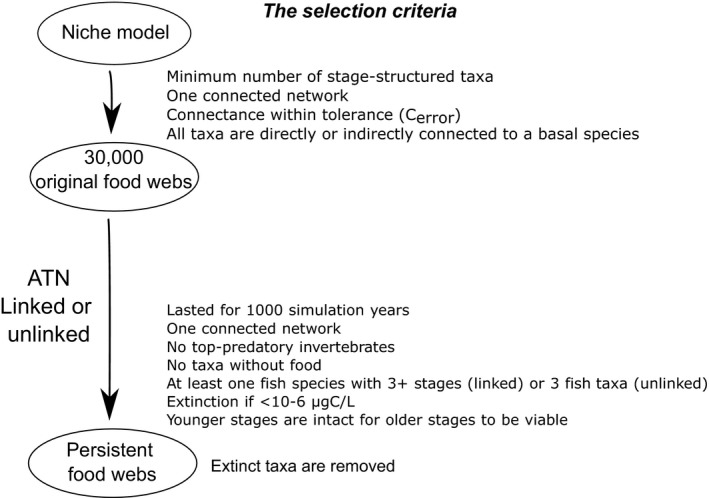
The list of the criteria used to select food webs for further analysis

#### 2.2.2 Dynamical model

The population dynamics within the food webs were formulated as a multispecies consumer–resource model (Bland et al., 2019; Brose et al., 2006; Williams & Martinez, 2004b; Yodzis & Innes, 1992). They were described by a set of ordinary differential equations (ODE).

(1) $$\begin{array}{c} \frac{dB_{i}}{\mathrm{dt}}=\overset{\text{logistic growth of autotrophs}}{\overbrace{g_{i}\left(1‐\sum_{j\in \text{autotrophs}}\frac{B_{j}}{K}\right)B_{i}}}‐\overset{\text{loss to grazing}}{\overbrace{\sum_{j\in \text{consumers}}x_{j}y_{\mathrm{ji}}B_{j}\frac{F_{\mathrm{ji}}}{e_{\mathrm{ji}}}}}\\ \frac{dB_{i}}{\mathrm{dt}}=\underset{\text{metabolic loss}}{\underbrace{‐f_{m}x_{i}B_{i}}}+\underset{\text{dietary intake}}{\underbrace{\sum_{j\in \text{resources}}f_{a}x_{i}y_{\mathrm{ij}}B_{i}F_{\mathrm{ij}}}}‐\underset{\text{loss to predation}}{\underbrace{\sum_{j\in \text{consumers}}x_{j}y_{\mathrm{ji}}B_{j}\frac{F_{\mathrm{ji}}}{e_{\mathrm{ji}}}}} \end{array}$$

where *g_i_* was the intrinsic growth rate of autotroph *i*, *K* was the carrying capacity, *x_i_* was the metabolic rate of consumer *i*, *y_ij_* was the maximum consumption rate relative to metabolic rate, *e_ij_* was the assimilation efficiency of predator *i* eating prey *j*, *f_m_* was the fraction of assimilated carbon lost for maintenance, and *f_a_* was the fraction of assimilated carbon that contributes to biomass growth (see Table 1b for parameter values). The model deterministically simulated the biomass dynamics during growing seasons. *F_ij_* was the functional response of consumer *i* when dealing with prey *j*

$$F_{\mathrm{ij}}=\frac{\frac{\omega_{\mathrm{ij}}}{\sum_{l\in \mathrm{resources}}\omega_{\mathrm{il}}}B_{j}^{q}}{B_{0_{\mathrm{ij}}}^{q}+\sum_{k\in \mathrm{consumer}}\left(c_{\mathrm{kj}}p_{\mathrm{ik}}B_{k}B_{0_{\mathrm{ij}}}^{h}\right)+\sum_{l\in \mathrm{resources}}\left(\frac{\omega_{\mathrm{ij}}}{\sum_{l\in \mathrm{resources}}\omega_{\mathrm{il}}}B_{l}^{q}\right)}$$

where $\omega_{\mathrm{ij}}$ was the preference of consumer *i* toward prey *j*, $B_{0_{\mathrm{ij}}}$ was the half saturation density for consumer *i* eating prey *j*, $c_{\mathrm{kj}}$ was the predator interference competition coefficient of *k* eating *j*, and *p_ik_* was the fraction of resources of consumer *i* shared with consumer *k*. The values of $B_{0_{\mathrm{ij}}}$ and $c_{\mathrm{kj}}$ varied among taxa and were taken from Boit et al. (2012) and Bland et al. (2019, their Figure 1) with modifications (Table 1; also see Martinez et al., 2012; Tonin, 2008). The parameters for interspecific or between‐stage interference competition were set to zero (i.e., $c_{\mathrm{kj}}=0$ for k≠i) for simplicity (sensitivity to these assumptions was checked in the sensitivity analysis). Previous studies that used the ATN framework for aquatic systems (Bland et al., 2019; Boit et al., 2012; Brose et al., 2006) differentiated the assimilation rates of consumers between nonbasal and basal species only. We added a rate for fish prey because fish is highly effective food for fish growth (Table 1; Juanes et al., 2002; Post, 2003) and lowered the assimilation rate for nonbasal species (i.e., invertebrates) to have the average of the two rates remain the same.

We added an ecologically plausible assumption that fishes preferred to feed on fish over invertebrates and on invertebrates over autotrophs, if they were included in their diets, to quickly grow beyond a size vulnerable to predation and for higher fecundity. To achieve these preferences in the absence of such empirical data, we set the parameter $\omega_{\mathrm{ij}}$ such that fishes whose diets included both autotrophs and animals fed almost exclusively on fish, to a lesser extent on invertebrates, but not much on autotrophs (Table 1b). Similarly, we assumed that invertebrates preferred invertebrates over fish and autotrophs. Growth of fish depends on the quantity and quality of food they eat, and shifting to piscivory invariably increases fish growth rate (Jobling, 2002; Juanes et al., 2002; Persson, 2002). As fish grow, piscivory could be necessary to meet energetic demands (Juanes et al., 2002). Also, because optimal morphologies for different diets (e.g., planktivory, benthivory, piscivory) are quite different, trade‐offs often arise and a diet specializing on the most profitable is likely preferred (Persson, 2002). Herbivory by fish occurs mostly in tropics and is much less common above 55° latitude because the enzyme to digest plant material is not active at low temperatures (González‐Bergonzoni et al., 2012; Vejříková et al., 2016). If we assumed no preference of fish for prey items (consumption proportional to relative availability), the majority of fish would consume high proportions of autotrophs due to their high abundance, an unlikely scenario in temperate and northern systems. If prey taxa went extinct (<10^–6^), they were removed from preference calculation.

The Hill exponent *q* of the functional response was set to 1.5, at the higher end of the values commonly used in previous ATN models (1.2–1.5), to ensure sufficient dynamical stability in large food webs (see Figure A5 for sensitivity analysis; Williams & Martinez, 2004b). The high value of the exponent was desired especially because food preferences of consumers increased energy flow higher up in the food web and reduced stability of the food webs in the model (Martinez et al., 2006). Higher values of *q* effectively converted the functional response closer to Holling type III (*q* = 2), which implicitly incorporates prey refugia, other evasive behavior, or adaptive foraging (Koen‐Alonso & Yodzis, 2005; McCann, 2000).

#### 2.2.3 Growth and reproduction

Growth and reproduction from surplus energy (dietary intake – metabolic loss – loss to predation; Equation 1) were accounted for at the end of the growing season when the ODE model was paused, which implicitly assumed that fishes all reproduced at the beginning of each growing season (Bland et al., 2019; Kuparinen et al., 2016). The fraction of mature fish in each stage was determined by using a logistic function (Table 2). We assumed that 50% of individuals were mature halfway through to the terminal stage. For example, if the taxon had five stages, about 50% of individuals were mature in Stage 3. We further assumed that fish in immature stages invested all their surplus energy in somatic growth, while mature fish allocated surplus energy to both growth and reproduction (Kuparinen et al., 2016). The allocation to reproduction (*I*) linearly increased with stage, and the terminal stage allocated 20% of surplus energy to reproduction (Table 2). Therefore, the biomass of the first stage class produced through reproduction was surplus energy multiplied by the probability of being mature and reproductive investment. We used the Leslie matrix to shift somatic biomass to the stage above via growth and to convert it to new recruitment (Table 2). The model allowed phenotypic variability within a stage such that some individuals did not grow enough during the preceding growing season to be recruited to the higher stage. We assumed that fish in the terminal stage additionally reproduced in exchange for somatic mass (Wootton, 1998). Each column added up to 1 in this formulation; therefore, there was no loss of biomass between consecutive growing seasons (i.e., fish did not gain or lose mass or die during winter).

### 2.3 Simulation design

We generated 10,000 networks of 60 nodes and connectance equal to 0.15 containing between 2 and 6 stage‐structured fishes with at least 3 and up to 5 stages (Table 1a). We generalized the fixed numbers used by Bland et al. (2019; three fish species with three stages). Food web studies on northern temperate systems typically include two to three species of fish, each of which has two to four stages (e.g., Boit et al., 2012; Claessen et al., 2002; Kuparinen et al., 2016; de Roos et al., 2008; Uusi‐Heikkilä et al., 2018) The minimum overlap between feeding ranges to be qualified as consecutive stages of the same stage‐structured fish species was 20% (Amundsen et al., 2003; Persson, 1983; Rezsu & Specziar, 2006). We then ran the ATN on each network from random initial biomasses uniformly distributed between 0.1 and 100 μgC/L. We ran two sets of simulations on the same networks. In one set, stages were linked via growth and reproduction as described above (“linked”), while in the other set, we removed the growth and reproduction links between stages (“unlinked”) to examine the effects of the additional biomass flow on the resulting food web dynamics and persistence. The simulations were otherwise identical (including the seed for the random number generator). Taxa were considered extinct when the biomasses became < 10^–6^ μgC/L (Table 1b), which was many orders of magnitudes smaller than the mean total fish biomass (10^1.1^ μgC/L). At the end of each generation, fishes that retained only older stages but not younger ones for more than 10 generations were removed as extinct. This happened in some simulations because the biomasses of older stages without younger ones lingered although they were to decay over time. Each year consisted of 90 time‐steps, representing one growing season, followed by a nongrowing season where reproduction and growth were accounted for. One time‐step corresponded to the generation time of the autotrophic species (i.e., *r* = 1; Boit et al., 2012; the growth rates of other taxa were normalized to the time scale of the basal species, as typically done in the ATN models; Brose et al. 2006). Food webs were regarded as persistent if simulations lasted for 1,000 growing seasons, which was sufficient for transient dynamics to die out for most of the simulation runs, with all the nodes of persisting species connected in one network, no invertebrates without predators, no species without food (i.e., transients did not completely die out in a handful of webs), and at least one fish with 3 or more stages for the linked case or at least 3 fish taxa for the unlinked case. We note that we regarded food webs as persistent if they retained fishes and met other criteria we just stated (Figure 3) to the end of the simulations, not by the proportions of taxa (nodes) persisted as typically reported by food web studies.

### 2.4 Analyzing model outputs

We first assessed various food web properties of the 10,000 networks (“original webs”) to check whether the generated food webs were reasonable. Then, we identified the linked and unlinked food webs that persisted for 1,000 growing seasons (i.e., years) and passed the abovementioned criteria (Figure 3), and retained only persisting taxa in these webs (“ATN‐filtered webs”). We then examined food web properties of the ATN‐filtered food webs using the last 100 years of the simulations. We calculated the mean of the CVs of biomasses of fish stages to characterize dynamical stability of food webs and the number of nodes and link density to characterize food web complexity. We also compared the unlinked and the linked webs for total fish biomass, the mean body masses of fishes weighted by relative abundance, the number of fish stages, and the maximum trophic levels and median predator–prey body mass (PPMR) ratios for fish stages. We computed a surrogate of total interaction strengths by summing up all energy flows into fish stages from their resources, CVs and skewness of the energy flows, which were individually normalized by the total gain of the recipient fish (Gross et al., 2009), and the slopes of biomass spectra by fitting a linear model to estimate biomasses at trophic levels as a function of body mass (Trebilco et al., 2013). The presence of weak interactions is theorized to stabilize food webs (McCann, 2012; McCann et al., 1998). Less negative slopes of biomass spectra indicate less bottom‐heavy food webs (Trebilco et al., 2013), which tend to be less dynamically stable than more bottom‐heavy food webs (McCauley et al., 2018; Rip & McCann, 2011). Some measurements were log_10_ transformed for interpretability. We computed 10,000 bootstrap estimates of the mean effect sizes of a stage structure (linked – unlinked) for these metrics by taking the differences between the means of resampled values from persisting linked and unlinked food webs.

We checked the sensitivity and robustness of the model outputs to major input parameters (Table 1). We ran 3,000 simulations for each parameter variation (17 sets in total) using the same food web topologies as the baseline simulations. We computed 1,000 bootstrap estimates of the mean effect sizes of a stage structure (linked – unlinked) for these metrics by taking the differences between the means of resampled values from persisting linked and unlinked food webs and compared the mean effect sizes with those from the baseline simulations.

## 3 RESULTS

### 3.1 Structural properties of the stage‐structured food webs

**FIGURE A3:**
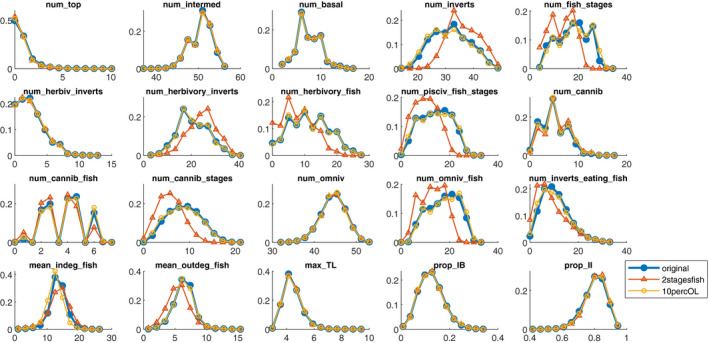
The frequency histograms of the network and food web properties measured on the 10,000 original food webs (original = not ATN‐filtered). Also shown are original webs from two sensitivity runs (2stagesfish = where the min and max numbers of fish stages are reduced from 3 and 5 to 2 and 4, respectively. 10percOL = where the overlap between adjacent fish stages is reduced from 20% to 10%). The *x*‐axes are the values of the metrics in the titles. The full names and definitions of these metrics are in Table A1

The structural properties and characteristics of the 10,000 original stage‐structured food webs generated by the modified niche model were measured on the unweighted networks (Figure A3). The majority of the 60 nodes were invertebrates, followed by 6–30 life‐history stages of fishes (2–6 species as specified) and 3–12 autotrophs. Between 6 and 30 fish stages were piscivorous (their diets included fish), while none to 28 of them had autotrophs in their diets. Fishes were almost all cannibalistic at some stage. Omnivores (feeding at 2 or more trophic levels) were abundant (40–50 species). Each fish stage had about 14 prey species and 7 predators on average. The mean maximum trophic level was near 5. Almost all interactions involved intermediate taxa (taxa with both prey and predators). About a half (4,937) of the webs did not have a top predator (taxa without a predator), while another 34% had one and 10% had two top predators. The sensitivity analysis showed that these patterns remained almost identical when ${\mathrm{OL}}_{\min}$ was reduced to 10%, while the number of fish stages decreased along with other measures as a direct consequence of having fewer fish stages when ${\mathrm{NStage}}_{\min}$ and ${\mathrm{NStage}}_{\max}$ were decreased (Figure A3).

### 3.2 The impact of a stage structure on the food webs

**FIGURE A4:**
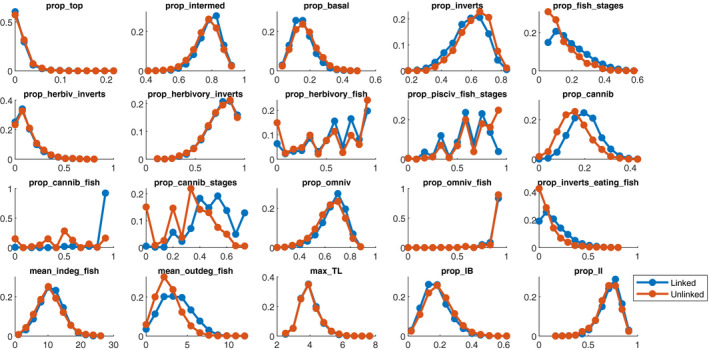
The frequency histograms of the network and food web properties measured on the 4,315 linked (blue) and 2,857 unlinked (red) persisting food webs. The *x*‐axes are the values of the metrics in the titles. The full names and definitions of the metrics are in Table A1

More than 1.5 times as many of the linked food webs persisted to the end of the simulations (i.e., 1,000 growing seasons) as the unlinked webs. Out of 10,000 food webs, 4,315 linked and 2,857 unlinked webs persisted for 1,000 growing seasons and passed the criteria, of which 2,461 webs were shared. Assessed by a set of network structural metrics and characteristics quantified on the unweighted networks after removing extinct taxa (“ATN‐filtered”; note that taxa that went extinct could be different between the linked and unlinked webs although the networks were identical to start with), the persisting food webs in both scenarios largely overlapped in terms of the majority of the properties measured (Figure A4). The number of persisting nodes ranged from 10 to 60, of which invertebrates comprised the majority and 3–29 nodes were life‐history stages of fishes. About a half (4,937) of the linked food webs did not have a top predator.

**FIGURE 4:**
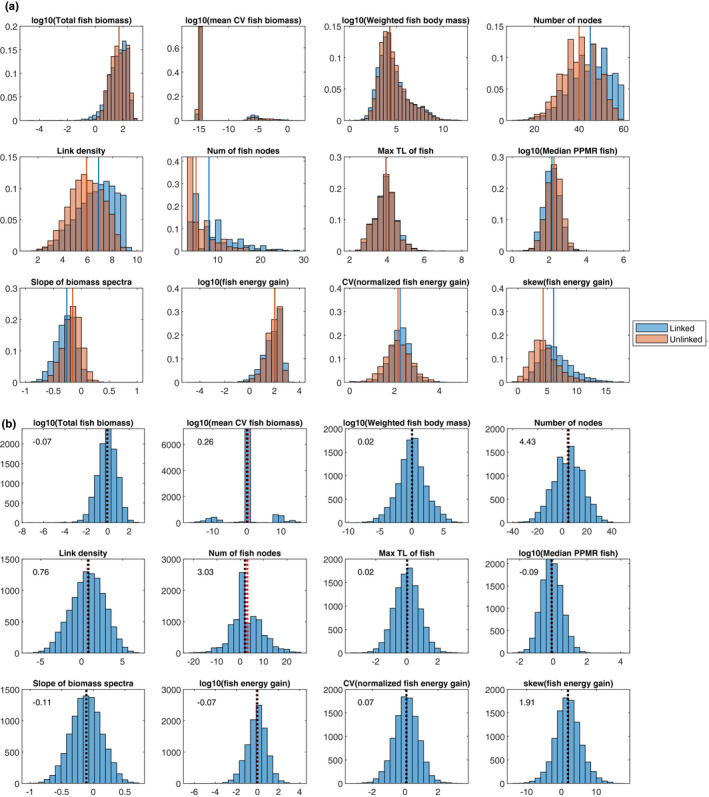
(a) The frequency distributions of the values of the 12 metrics measured on the 4,315 linked (blue) and 2,857 unlinked (orange) persisting webs. The lines in the corresponding color indicate the locations of the means. The values are average across the last 100 years of the 1000‐year simulations. Total fish biomass = the sum of biomasses of all fish stages, mean CV fish biomass = mean of the CVs of individual fish stages, weighted fish body mass = body masses of fish stages weighted by relative abundance, link density = the number of links divided by the number of nodes, max TL of fish = maximum trophic level of fish stages, median PPMR fish = median predator–prey mass ratio for fish stages, fish energy gain = total energy entering fish stages, normalized fish energy gain = energy flow through individual links into fish stages divided by the total energy gained by the recipient fish stages (see the explanation in the text), skew(fish energy gain) = skewness of energy flow through individual links into fish stages. (b) The frequency distributions of the bootstrap differences in means between the linked and unlinked food webs (linked – unlinked) in terms of the 12 metrics. The black dotted lines indicate the locations of the means, and the numbers at the top left corner are the means

The distributions of measured food web properties between the linked and unlinked food webs overlapped substantially (Figure 4a). Nonetheless, the bootstrap differences in means between the linked and unlinked food webs (linked – unlinked webs) highlighted that some of the mean effect sizes were noticeable; the number of nodes was greater by 4.3, the slope of biomass spectra was 0.11 smaller, and the skewness of normalized fish energy gain was 1.9 larger in the linked webs than in the unlinked webs on average (Figure 4b). These suggested that, when stages were linked relative to when unlinked, on average the biomass pyramids were more bottom‐heavy, food webs sustained several more species or fish stages, and fish energy gain was more variable among links and dominated by small gains (indicated by the positive skews).

**FIGURE A5:**
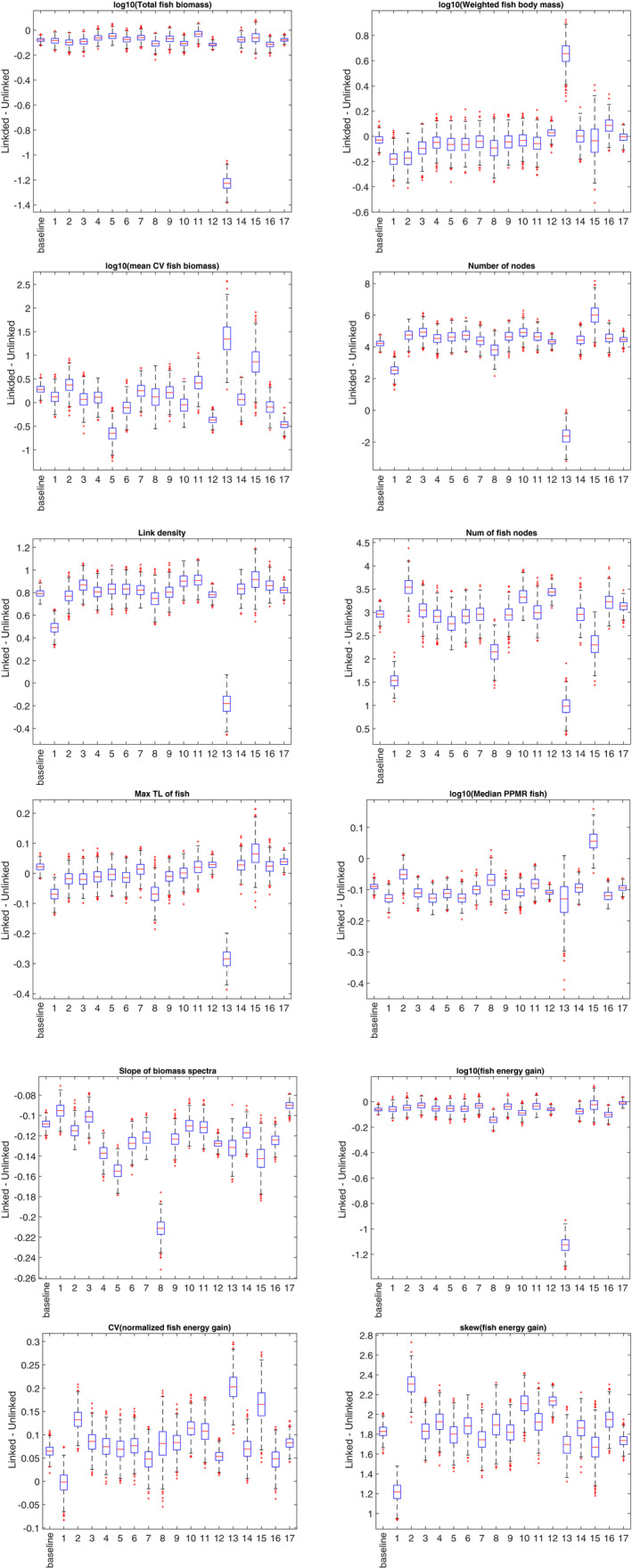
Distributions of 1,000 differences between sample means of linked and unlinked persisting webs randomly selected with replacement for the baseline simulations (presented in the maintext) and 17 simulation sets for sensitivity analysis. The data shown in the baseline column are the same as those in Figure 4. The numbers below the figures correspond to the numbers given in the last columns of Table 1a and b in the main text. Total fish biomass = the sum of biomass of all fish stages, mean CV fish biomass = mean of the CVs of individual fish stages, weighted fish body mass = body mass of fish stages weighted by relative abundance, link density = the number of links divided by the number of nodes, max TL of fish = maximum trophic level of fish stages, median PPMR fish = median predator–prey mass ratio for fish stages, fish energy gain = total energy entering fish stages, normalized fish energy gain = energy flow through individual links into fish stages divided by the total energy fish energy gain (see the explanation in the text), and skew(fish energy gain) = skewness of energy flow

Taken together, the results suggested that the life‐history stage structure of fishes on average supported higher diversity in these complex food webs and promoted characteristics that potentially increase the stability of food webs, which may explain the greater number of persisted food webs when fish stages are linked than unlinked. The sensitivity analysis showed that the patterns in the bootstrap differences in mean effect sizes of having a stage structure of fishes remained largely in the same direction (negative or positive) as the baseline simulations across the sensitivity analysis simulations except several cases (i.e., the effect sizes were all positive for the number of nodes except #13 and for the skewness of fish energy gain; they were all negative for the slope of biomass spectra; Figure A5). The bootstrap differences qualitatively deviated from the baseline runs for some measures when the maximum and minimum numbers of fish stages $\left({\mathrm{NStage}}_{\max},\;{\mathrm{NStage}}_{\min}\right)$ were smaller (#1), the minimum overlap of niche ranges between consecutive stages of a fish was reduced to 10% (#2), fishes were less energetically efficient (#8), all consumer species and fish stages underwent interference competition (#13), and when *q* was reduced to 1.2 (#15). The number of persisting webs was markedly smaller (17.8% when linked, 12.7% when unlinked) when *q* = 1.2 (data not shown).

## 4 DISCUSSION

### 4.1 The modified niche model

To introduce life‐history stages into food webs, we developed additional algorithms that modified food webs from the niche model by Williams and Martinez (2000). Food web topologies generated by other food web structural models can also be used as long as they assign feeding hierarchies and ranges to every taxon, such as the variants of the niche model (generalized niche model (Stouffer et al., 2006); the relaxed niche model (Williams & Martinez, 2008); the minimum potential niche model (Allesina et al., 2008)). Our method utilized the concepts of ontogenetic diet shifts and niche overlap among ontogenetic stages (Werner, 1986; Werner & Gilliam, 1984) to identify life‐history stages and heuristically assembled the specified number of taxa with a stage structure. It can be adapted to other situations where, for example, stage‐structured taxa feed higher in the trophic level (e.g., set the minimum trophic level > 3 to become a fish or exclude consumers feeding on autotrophs from the pool of fish candidates) or feeding range overlaps are smaller or larger. The outputs can be fed into the ATN framework or other dynamical models that can accommodate biomass flow via growth and reproduction.

We grouped trophic species created by the niche model to assemble a stage‐structured fish taxon, unlike the previous models where a trophic species was split into stages (Bland et al., 2019; Rudolf & Lafferty, 2010). As a result, our method generated food webs that largely preserved topologies (i.e., besides removing rare within‐stage and reverse cannibalism) produced by the niche model, which has been shown to reproduce empirically observed food web properties (Williams & Martinez, 2000, 2008). The previous methods introduced new nodes and links, likely compromising the merit of using the niche model. Our approach also agrees with the method employed by Williams and Martinez (2000, 2008) to evaluate the niche model's performance, where some of the empirical data they used distinguished different stages of the same species (e.g., larval/young‐of‐year and adult fish in Little Rock Lake, Ythan estuary, and Chesapeake data). Our approach hence followed from the definition of trophic species, a group of taxa sharing predators and prey, from the common phenomena of the ontogenetic diet shift, and from the fact that the niche model creates trophic species. What constitutes a trophic species should depend on the level of aggregation appropriate for a given study. Because we were interested in trophically distinct roles of ontogenetic stages on food web dynamics (Werner, 1986), it was both convenient and reasonable to interpret trophic species as ontogenetic stages and group multiple trophic species into a stage‐structured species. It appears to be a great advantage to minimize alteration of food webs obtained from the niche model. As a by‐product, we also eliminated the convoluted steps to assign niche values to newly created nodes in the method by Bland et al. (2019). We think that our approach improves and simplifies their method, making it more conceptually accessible to food web researchers.

In our results, a greater number of food webs with linked stages persisted than those with unlinked stages, although linked stage‐structured food webs were qualified with more stringent criteria (namely, higher stages cannot persist without lower stages for more than 10 generations vs. independent stages; at least one fish with 3 or more stages persisting vs. at least any 3 fish nodes persisting). Once persisting, food webs with linked life‐history stages were more complex, indicated by the higher numbers of persisting taxa (nodes) and higher link density (Figure 4). The relative frequency of food webs with oscillating biomass dynamics (i.e., higher CV) was similar between linked and unlinked webs (Figure 4a), which was also observed in Bland et al. (2019). The linked webs contained more weak links than the unlinked webs, as the mean of the skewness of individual energy flow into fishes was modestly higher in the linked webs on average. Having weak interactions is one of the key properties that can increase stability of food webs (Gellner & McCann, 2016; McCann, 2000, 2012; McCann et al., 1998). Furthermore, we observed that the linked webs had lower slopes of biomass spectra and hence exhibited more bottom‐heavy biomass pyramids than did the unlinked webs. Bottom‐heavy biomass pyramids tend to relate to dynamically stable consumer–resource dynamics, while top‐heavy biomass pyramids tend to suggest unstable dynamics (McCauley et al., 2018; Rip & McCann, 2011; Trebilco et al., 2013). Therefore, the stabilizing effects of life‐history stages that we saw in our simulations appear in agreement with what current food web theories predict.

Our method and the method by Bland et al. (2019) also differed in modeling demographic shifts via growth and reproduction at the end of growing seasons. The differences were in how surplus energy was dealt with and in the proportion of the biomass of the terminal fish stage to be transferred to the first stage. Thus, the differences between our results and those of Bland et al. (2019) may not be attributable only to how life‐history stages were constructed (grouping nodes versus. splitting a node). Further research should systematically explore how a life‐stage structure can affect food web stability. Our method can serve as a tool to generate biologically justifiable stage‐structured food web topologies to facilitate such explorations in future studies.

The original niche model by Williams and Martinez (2000) produces many consumers that include autotrophs in their diets. In temperate and northern regions, fishes feeding on autotrophs are uncommon because of low activity levels of digesting enzymes (González‐Bergonzoni et al., 2012; Vejříková et al., 2016). We controlled the consumption of autotrophs through prey preferences of consumers in the dynamic model. As a result, fishes consumed little autotrophic biomass (mean = 8% in the baseline simulations) in the simulations, despite including autotrophs in their diets.

### 4.2 Life‐history structure and food web stability

How a life‐history structure may affect the stability and persistence of complex food webs has not been much studied. It is not immediately clear whether it is stabilizing or destabilizing based on existing theories. We can expect multiple aspects of stage‐structured populations to contribute to instability. As discussed by Rudolf and Lafferty (2010), when stage classes have smaller subsets of the feeding range than the species as a whole, resources essentially become less substitutable, especially when the overlaps between feeding ranges are small. Thus, if resources for one stage become scarce, the persistence of the entire species is greatly endangered unless growth and reproduction can constantly replenish the dwindling stage. Similarly, as stages become more specialized, consumer–resource interactions may become less diffuse and some of the remaining interactions may strengthen. Because weak interactions tend to stabilize trophic interactions (Gellner & McCann, 2016; McCann, 2012), specialized stages likely reduce stability of food webs. Also, a stage structure introduces delays and asymmetry between stages into population models, both of which are known to often cause population instability in the forms of cohort cycles and alternative stable states (Gellner et al., 2020; McCann, 2012; de Roos & Persson, 2013). Therefore, the odds seem to be against increased food web stability by introducing life‐history stages.

Stages in structured populations can subsidize dwindling stages through growth and reproduction, which is probably one of the main reasons why a stage structure in food webs could enhance the persistence of stage‐structured populations and other dependent populations. Furthermore, biomass flow via growth or reproduction between competing stages with overlapping diet might moderate the destabilization effects of exploitative competition. Stouffer and Bascompte (2010) showed that the exploitative competition module reduced food web persistence as it increased in frequency in dynamical models of complex food webs. This effect of diet overlap may appear contradictory to the result from Rudolf and Lafferty (2010), which showed that, when feeding niches were overlapping by more than about 30%, the inclusion of stages increased the robustness of food webs. Because they studied the robustness of static food webs (only topology, no dynamics), diet overlap reduced reliance of a stage‐structured population on any particular resource, as the authors explained. In dynamic models, exploitative competition can ensue and drive one of the competitors and possibly other populations to extinction, but if the competitors are ontogenetic stages of the same species, biomass flow between the stages could alleviate competitive exclusion. In the study by Stouffer and Bascompte (2010), the frequency of the tritrophic food chain module had positive effects on persistence in large food webs. In a sense, life‐history structured populations contain a biomass flow chain inside. We conjecture that this might also contribute to food web stability. In addition to the possible adverse effects on stability we discussed above, ontogenetic asymmetry may also help some populations persist in food webs. de Roos et al. (2008) showed in stage‐structured food web modules that persistence of consumers could be promoted in communities with stage‐structured prey through emergent facilitation due to biomass compensation in the prey population. It seems reasonable to state that the effects of life‐history stages on the stability of complex food webs are complex and contingent on the balance of the effects of different processes.

## 5 CONCLUSIONS

Life‐history stages constitute part of biological diversity and increase complexity of food webs. As a large majority of organisms grow in size, often over orders of magnitude, during their lifetime and experience various degrees of ontogenetic diet shifts (Werner & Gilliam, 1984), life‐history structures are important to be considered in studies on the stability of complex food webs. In this study, we demonstrated a positive relationship between the complexity and stability of complex food webs; food webs with stage‐structured taxa persisted more often and supported more taxa than did those with unlinked stages. These results are qualitatively in agreement with the findings by Mougi (2017). For aquatic systems and fishes in particular, ontogenetic stages are well recognized and studied so that including life‐history stages explicitly in models can facilitate linking theory and data. Practically, including separate stages makes it more mechanistic and straightforward to implement allometrically scaled functions or parameters and differences in behaviors among stages. For example, simulating size‐selective fishing and the evolutionary impacts of such fishing on the population dynamics of exploited species in food webs becomes more straightforward once a life‐history structure is explicitly incorporated (e.g., Kuparinen et al., 2016). Moreover, our work contributes a way of incorporating another aspect of interaction diversity via life histories to the growing research on multilayered networks (Kéfi et al., 2012, 2017). Biomass flow via growth and reproduction forms networks of energy transfer analogously to consumer–resource interactions. Research on multilayered networks has so far revealed that nontrophic interactions (thus interaction diversity) can ameliorate or degrade the stability of trophic interactions and the persistence of species (reviewed by Kéfi et al., 2017). Interestingly, Sauve et al. (2014) showed that network structures known to stabilize mutualistic interactions became less effective when combined with trophic interactions in a multilayered network. Tritrophic food chain and omnivory modules have been shown to stabilize complex food webs (Stouffer & Bascompte, 2010), and it will be instructive to examine if they still do so when embedded in complex food webs including ontogenetic biomass flow.

## CONFLICT OF INTEREST

None declared.

## AUTHOR CONTRIBUTIONS

**Etsuko Nonaka:** Conceptualization (equal); data curation (lead); formal analysis (lead); investigation (lead); methodology (lead); project administration (lead); visualization (lead); writing–original draft (lead); writing–review and editing (lead). **Anna Kuparinen:** Conceptualization (equal); formal analysis (supporting); funding acquisition (lead); investigation (supporting); methodology (supporting); project administration (supporting); resources (lead); software (lead); supervision (lead); writing–review and editing (supporting).

## Data Availability

The MATLAB code for the modified niche model and the ATN model used in this study is available in the Dryad Digital Depository https://doi.org/10.5061/dryad.z612jm6bk

## APPENDIX 1

**TABLE A1:** Network metrics used in this study and their definitions

Name	Definition
In Figure 4	
Total fish biomass	Sum of biomasses of all fish stages
Mean CV fish biomass	Mean of the CVs of individual fish stages
Weighted fish body mass	Body masses of fish stages weighted by relative abundance
Link density	Number of links divided by the number of nodes
Max TL of fish	Maximum trophic level of fish stages
Median PPMR fish	Median predator–prey mass ratio for fish stages
Fish energy gain	Sum of energy entering fish stages
Normalized fish energy gain	Amount of energy flowing through individual links into fish stages divided by total energy gained by the recipient fish stages
Skew(fish energy gain)	Skewness of the amounts of energy flowing through individual links into fish stages
In Figure A3	
num_spp	Number of trophic species (nodes)
num_top	Number of taxa without predators
num_intermed	Number of intermediate species (having both prey and predators)
num_basal	Number of autotrophs
num_inverts	Number of invertebrates
num_fish_stages	Number of fish stages
num_herbiv_inverts	Number of herbivorous invertebrates
num_herbivory_inverts	Number of invertebrates that include autotrophs in the diet
num_herbivory_fish	Number of fish stages that include autotrophs in the diet
num_pisciv_fish_stages	Number of fish stages that include fish in the diet
num_cannib	Number of cannibalistic taxa
num_cannib_fish	Number of cannibalistic fish species
num_cannib_stages	Number of cannibalistic fish stages
num_omniv	Number of omnivorous taxa (feeding at multiple trophic levels)
num_omniv_fish	Number of omnivorous fish stages
num_inverts_eating_fish	Number of invertebrates that eat fish
mean_indeg_fish	Mean number of prey taxa of fish stages
mean_outdeg_fish	Mean number of predator taxa of fish stages
max_TL	Maximum trophic level
prop_IB	Proportion of interactions involving an intermediate taxon and an autotroph (basal)
prop_II	Proportion of interactions involving two intermediate taxa
In Figure A4	
prop_top	Proportion of taxa without predators
prop_intermed	Proportion of intermediate taxa (having both predators and prey)
prop_basal	Proportion of autotrophic taxa
prop_inverts	Proportion of invertebrates
prop_fish_stage	Proportion of fish stages
prop_herbiv_inverts	Proportion of herbivorous invertebrates
prop_herbivory_inverts	Proportion of invertebrates that include autotrophs in the diet
prop_herbivory_fish	Proportion of fish stages that include autotrophs in the diet
prop_pisciv_fish_stages	Proportion of fish stages that include fish in the diet
prop_cannib	Proportion of cannibalistic taxa
prop_cannib_fish	Proportion of cannibalistic fish species
prop_cannib_stages	Proportion of cannibalistic fish stages
prop_omniv	Proportion of omnivorous taxa (feeding at multiple trophic levels)
prop_omniv_fish	Proportion of omnivorous fish stages
prop_inverts_eating_fish	Proportion of invertebrates that eat fish
